# Impact of telehealth interventions on physiological and psychological outcomes in breast cancer survivors: A meta-analysis of randomised controlled trials

**DOI:** 10.3389/fonc.2022.1017343

**Published:** 2023-01-05

**Authors:** Puneeta Ajmera, Mohammad Miraj, Sheetal Kalra, Ramesh K. Goyal, Varsha Chorsiya, Riyaz Ahamed Shaik, Msaad Alzhrani, Ahmad Alanazi, Mazen Alqahtani, Shaima Ali Miraj, Sonia Pawaria, Vini Mehta

**Affiliations:** ^1^ Department of Public Health, School of Allied Health Sciences, Delhi Pharmaceutical Sciences and Research University, New Delhi, India; ^2^ Department of Physical Therapy and Health Rehabilitation, College of Applied Medical Sciences, Majmaah University, AlMajmaah, Saudi Arabia; ^3^ School of Physiotherapy, Delhi Pharmaceutical Sciences and Research University, New Delhi, India; ^4^ Department of Pharmacology, Delhi Pharmaceutical Sciences and Research University, New Delhi, India; ^5^ Department of Family and Community Medicine, College of Medicine, Majmaah University, Al Majmaah, Saudi Arabia; ^6^ College of Applied Medical Sciences, AlMaarefa University, Dariyah, Riyadh, Saudi Arabia; ^7^ Department of Public Health, College of Health Science, Saudi Electronic University, Riyadh, Saudi Arabia; ^8^ Faculty of Physiotherapy, SGT University, Gurugram, India; ^9^ Department of Public Health Dentistry, Dr. D.Y. Patil Dental College and Hospital, Dr. D.Y. Patil Vidyapeeth, Pune, India

**Keywords:** Breast Cancer, Neoplasm, Tele-health, Meta-analysis, Physiological outcomes, Psychological outcomes

## Abstract

**Introduction:**

The use of telehealth interventions has been evaluated in different perspectives in women and also supported with various clinical trials, but its overall efficacy is still ascertained. The objective of the present review is to identify, appraise and analyze randomized controlled trials on breast cancer survivors who have participated in technology-based intervention programs incorporating a wide range of physical and psychological outcome measures.

**Material and methods:**

We conducted electronic search of the literature during last twenty years i.e., from 2001 till August 10, 2021 through four databases. Standardized mean difference with 95% confidence interval was used.

**Results:**

A total of 56 records were included in the qualitative and 28 in quantitative analysis. Pooled results show that telehealth interventions were associated with improved quality of life (SMD 0.48, 95% CI 0.03 to 0.92, p=0.04), reduced depression (SMD -1.27, 95% CI =-2.43 to -0.10 p=0.03), low distress and less perceived stress (SMD -0.40, 95% CI =-0.68 to -0.12, p=0.005). However, no significant differences were observed on weight change (SMD -0.27, 95% CI =-2.39 to 1.86, p=0.81) and anxiety scores (SMD -0.09, 95% CI =-0.20 to 0.02, p=0.10) between the two groups. Improvement in health care competence and fitness among participants was also reported.

**Conclusion:**

Study concludes that telehealth care is a quick, convenient and assuring approach to breast cancer care in women that can reduce treatment burden and subsequent disturbance to the lives of breast cancer survivors.

## Introduction

Breast cancer is the most common diagnosed cancer in women (1) and accounted for 2.1 million diagnosed cases and an estimated 626,679 deaths worldwide in 2018 (2). Due to advancements in diagnostic techniques and therapeutic treatment during the last few decades, 5-year survival rate of breast cancer patients has exceeded 85 percent (3). “Breast Cancer survivors” is a term commonly used for women living with cancer since the inception (diagnosis) of the disease and for the balance of life (4). Once a woman acquires breast cancer and even if she is treated, a continuous interdisciplinary supportive care is desired (5–7). Majority of the women experience various psychological problems like anxiety, depression and perceived stress which are generally substantial and prolonged (8–10) and require considerable healthcare support that may help them overcome psychological barriers and perceive their situation more positively (11). Every woman plays multifaceted roles in any normal scenario. For women, whether it is job or household responsibilities it is difficult for her to manage a separate time slot for visiting the consultant and get guidance in person (12). Such circumstances consequently brought in demand for alternative provision for health care service delivery, which prioritize the technology guided tele-intervention to come into role (13, 14). The technology acts as a boon in such cases where they can use telehealth consultation or regime and be a part of any fitness protocol during the micro breaks of their already scheduled activities (15, 16). Digital technology guided tele-intervention though are “complex” but have the potential for outreach, cost effectiveness and accessibility in managing the health related issues for consultation and treatment purposes using various application and online web services (13, 17–19). This trend is facilitated more with the inculcation of digital technology of mobile, application and dependency on artificial intelligence (20).

Researchers have investigated the effectiveness of variety of telehealth intervention for breast cancer survivors in a range of domains like quality of life, mental health, nutritional aspects etc (13, 14, 21–23). Tele-interventions targeting various spectrum of ages of women in multiple aspects across diverse racial and cultural perspectives have been shown to be satisfactory to the end-user and realistic to implement (24, 25). Although the use of telehealth interventions have been evaluated in different perspectives in women and also supported with various clinical trials, but its overall efficacy is still ascertain due to difficulty in designing or implementing non-biased randomized controlled trials (RCT) exploring its true effect. A generalized search in data bases indicates that most of the reviews performed on breast cancer survivors has targeted only Quality of life and psychological outcome measures (13). There is a dearth of published systematic reviews on the impact of telehealth guided interventions on outcomes other than Quality of life and psychological measures in breast cancer survivors and that has formed the basis of this review. To the best of author’s knowledge, this meta-analysis is first of its kind to access the effectiveness of spectrum of telehealth interventions on a variety of clinical and psychological outcomes in breast cancer survivors.

The objective of the present review is to identify, appraise and analyze qualitative and quantitative research evidence for breast cancer survivors who have participated in technology based tele-intervention programs incorporating a wide range of physical, physiological and psychological outcome measures. The intent of the present systematic review will help in providing important consideration for potential outcome of telehealth guided tele intervention with a future insight on its successful uptake.

## Materials and methods

**Figure 1:**
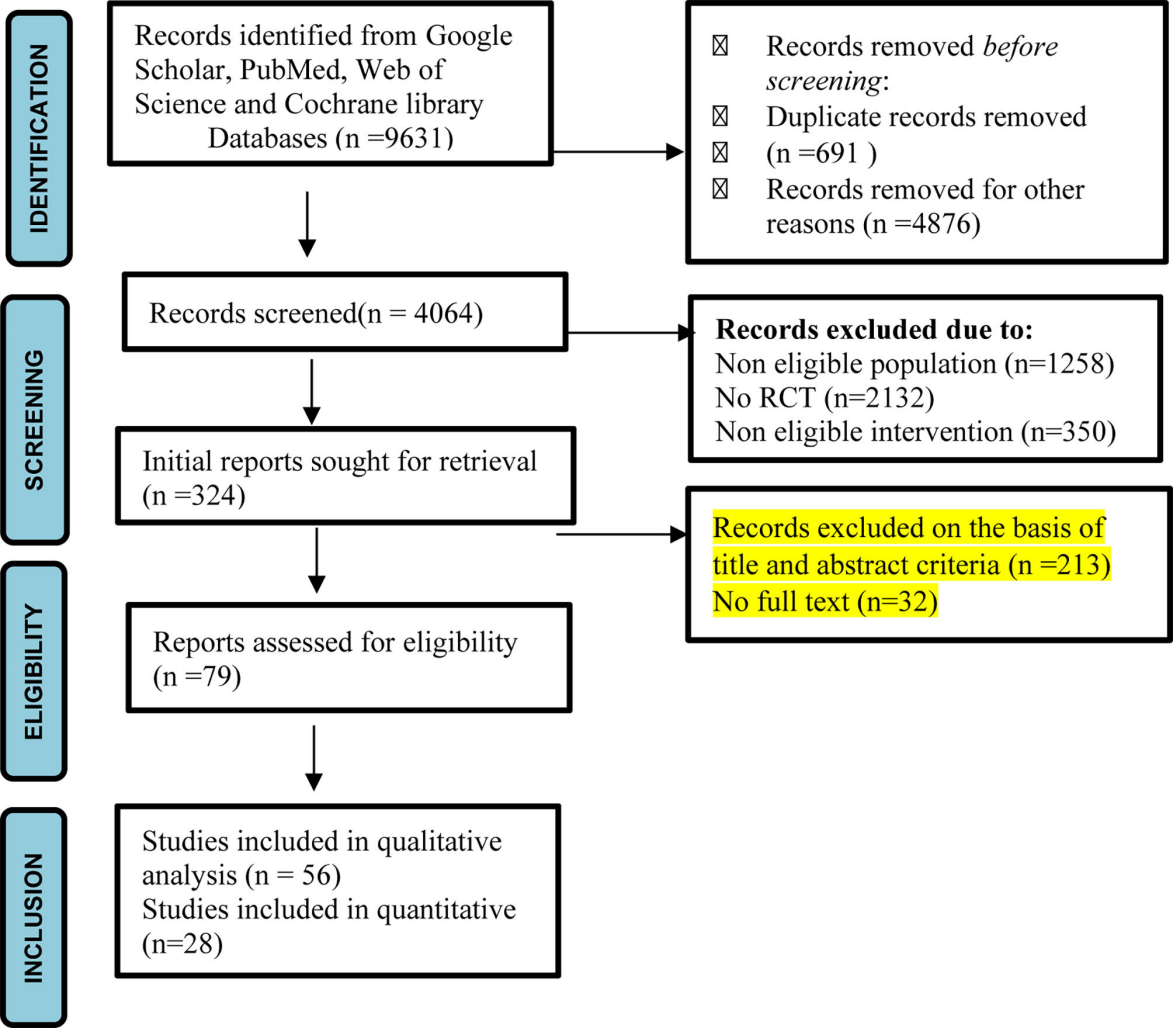
PRISMA flow diagram of study (27).

Preferred reporting items for systematic review and meta-analysis protocols (PRISMA)statement was used for to develop and report this systematic review (26) (**Figure 1**).

### Search strategy

We conducted electronic search of the literature during last twenty years i.e. from 2001 till August 10, 2021 through four databases viz. Google Scholar, PubMed, Web of Science and Cochrane library. To search more precisely, MeSH terms and Boolean operators were used in library databases. Search strategy used was: [Tele OR Tele health OR Tele technology OR Tele intervention OR Tele technologies OR Telemedicine OR Teleconsultation OR Telecommunication OR E health OR e Health OR Mobile Health OR mHealth OR Cell Phones OR Telephones OR Text Messaging OR SMS OR Videoconference OR Video-conference OR Videoconferencing OR Skype] AND [Breast cancer OR Breast neoplasm OR Breast cancer survivor OR Breast cancer survivor OR Breast neoplasm survivor OR Breast neoplasm survivors] AND [Woman OR Women OR Woman health OR Women health OR Health of Woman OR Health of Women].” To maximize literature coverage and cross check the results we followed multivaried methodology covering multiple databases. We used PICOS framework to select articles from the databases. *P* (Population) breast cancer patients. Telehealth intervention is compared to usual medical care alone in *I* (intervention) and *C* (comparison) respectively. Usual care referred to standard medical procedures such routine hospital visits for in-person treatment, conventional breast cancer education, and so on. *O* (Outcomes: Weight change, Quality of life and psychological outcomes, such as distress and perceived stress, anxiety, and depression. *S* (study design) only RCTs were included. Case reports, reviews, non-randomized controlled trials, duplicate reports, and studies with uninteresting data were excluded from consideration. PICOS framework is presented in **Table 1**.

**Table 1:** Search strategy - PICOS framework.

Framework	Search items
Population (P)	Breast cancer survivor OR Breast neoplasm survivor OR Breast neoplasm survivors OR Women diagnosed with breast cancer
Intervention (I)	(Tele OR Tele health OR Tele technology OR Tele intervention OR Tele technologies OR Telemedicine OR Teleconsultation OR Telecommunication OR E health OR e Health OR Mobile Health OR mHealth OR Cell Phones OR Telephones OR Text Messaging OR SMS OR Videoconference OR Video-conference OR Videoconferencing OR Skype)
Comparison (C)	(Usual care)
Outcome (O)	(Weight change, QOL and psychological outcome measures including depression, anxiety, distress and perceived stress.)
Study design	Randomized Controlled Trials

### Study selection

The process of eligibility was divided into subsequent phases with definite inclusion or exclusion criteria. Only full text academic articles published in peer-reviewed journals were included in the review whereas magazine and newspaper articles were excluded. Using the search strings, 324 papers from the four databases were identified in the phase, I. In phase II, duplicate papers in each search string and papers for which only abstracts were available were excluded. In the IIIrd phase, a new search category with papers impending under all established search strings was introduced and duplicates were removed across all search strings.

In phase IV, all full-length texts were thororghly assessed and papers that had no relevance to objectives and research questions of our study were excluded. Twenty Eight papers were finally selected and a descriptive analysis was executed to summarize the results.

### Inclusion and exclusion criteria

* Randomized Controlled Trials that examined the role of telehealth technologies in breast cancer survivors were included. Non randomized controlled trials, cross sectional studies, cohort and case control studies were excluded from the study.

* Full text articles written only in English language and published in peer-reviewed journals were included while articles in any other language, book chapters were excluded.

### Data extraction and management

Data was independently extracted by two reviewers, (SK) and (PA)on characteristics of study location, year of study, participants, study duration, sample size, inclusion and exclusion criteria, details of intervention, study duration, outcome measures and results of study. Wherever possible, post intervention mean scores and standard deviation were retrieved and recorded. Data was rechecked by third reviewer, (SP) and any discrepancy or doubt pertaining to the selection of particular study was resolved after exhaustive discussion among all the authors.

### Risk of bias

Risk of bias in individual studies and methodological quality assessment was performed by 2 independent reviewers SK and PA with more than 15 years of experience in empirical research. Cochrane collaboration tool was used to assess bias risk in randomized control trials in selected articles (28). The tool assesses bias risk on basis of 7 domains. The judgment regarding bias was categorized under 3 categories- a. Low risk b. High risk and c. unclear risk. PRISMA guidelines were used for reporting results of systematic reviews and Meta-analysis. Any disagreements between the 2 reviewers regarding appraisal recommendation were resolved by another reviewer (MM). Review Manager (RevMan) software version 5.4 is used for meta-analysis.

## Results

### Study selection search results

Initially, during literature search, 9631 records were identified from selected databases. During first screening 691 articles were removed due to duplication while 1258 records were removed as population was found to be non-eligible. Further 2132 records were non-RCTs and in 350 records intervention was not as per our eligibility, hence they were also removed. After initial screening, 324 titles emerged out to be relevant studies. After removal of duplicates and studies not fulfilling eligibility criteria, seventy nine full text records were identified and screened again. Fifty-six records were found to be relevant and directly within the scope of this review and therefore included in the qualitative analysis. Twenty three studies were included in quantitative analysis. Data was summarized narratively and descriptive analysis was carried out. Tables and graphs were prepared to convey significant features of the literature.

### Study characteristics

Fifty-six RCT’s met our inclusion criteria involving a total of 20,746 women. The earliest study meeting eligibility criteria was published in year 2001 (29). Thirty two trials were conducted in USA (22, 29–58), 7 in Australia (59–64), 4 in Netherland (65–68), 3 each in Denmark (14, 69, 70) and Spain (71–73), 2 in Germany (74, 75) and 1each in Turkey (76), Finland (77), Taiwan (42), Canada (78), UK (39) and Korea (79). Sample size ranged from 53 in the study of Owen et al, 2005 (76) to 3088 in the study of Pierce et al. in 2007 (30). The trials were conducted in different set ups ranging from cancer societies, multi center institutes, hospitals, medical centers, oncology clinics and Medical University. Age of Participants recruited in different studies ranged from minimum of 18 years to maximum of 80 years. Longest follow up of 4 years for events and mortality related to cancer was done by Pierce et al, 2007 (30). Characteristics of studies are shown in **Tables 2**, **3**. The types of technology used for telehealth interventions varied throughout the studies that were included. Twenty nine studies used telephone based interventions (22, 29–32, 34–36, 38–40, 42, 44–47, 49, 51, 52, 58–62, 66, 69, 75, 78, 80). Twelve studies used web based interventions (33, 47, 50, 53, 57, 63, 65, 68, 72, 74, 76, 77). Telemedicine was used in two studies (70, 73), eight studies utilized combination of Internet, web, telephone and videoconferencing (14, 37, 41, 43, 48, 56, 67, 71) where as in three studies wearable technology was used for weight management or physical activity tracking (33, 54, 64). Two studies used mobile health based app for self-management of symptoms and mobile gaming in cancer patients (42, 56).

**Table 2:** Characteristics of included trials.

S No.	Author/year/source	Country	Setting/Data Collection	Participants demographics/Age in years (Mean+SD)
1	Rock et al, 2001 (29)	USA	Multi-centre/started in 1995	N=1010 Age: Intervention group: 54.3± 0.4 Control group: 54.0 ± 0.4
2	Samarel et al, 2002 (31)	USA	Physician’s offices, hospitals, and the American Cancer Society/NM	N=125 30 to 83 years Age:53.8 ± 10.8
3	Pierce et al, 2004 (32)	USA	NM	N=2970 Mean Age:52 Years
4	Winzelberg et al, 2004 (33)	USA	NM	N=72 Age: 49.5 ± 6.2
5	Mishel, 2005 (34)	USA	Cancer centers and hospital based/NM	N=509 Age: 64 ± 8.9
6	Owen et al, 2005 (76)	Turkey	NM	N=53 Age: Intervention group: 52.5 ± 8.6; Control group 51.3 ± 10.5
7	Aranda et al, 2006 (59)	Australia	Hospital based	N=60 Age (Median/Range)Intervention group:57/ (34–85) Control group: 55/ (36–82)
8	Gotay et al, 2007 (35)	USA	Hospital based/ 1998-2002	N=305 Age: (Median/Range)Intervention group:53/ (34–93) Control group: 55/(25–90)
9	Pierce et al, 2007 (30)	USA	Multi-institutional/2000-2006.	N=3088 Age: Intervention group:53.3 ± 8.9; Control group:53.0 ± 9.0
10	Sandgren et al., 2007 (36)	USA	Oncology Clinics/NM	N=218 Age: (Mean) 54.4 years
11	Budin et al, 2008 (37)	USA	Medical Centers/NM	N=249 Age: 53.8 ± 11.7
12	Kathleen et al, 2009 (38)	USA	NM/2002-2004	N=487 Age-(Mean) Intervention group: 69 (55); Control group: 63 (55)
13	Beaver et al, 2009 (39)	UK	Outpatient clinic in hospital/2003- 2005.	N=374 Age: Intervention group:64.0 ± 11.1; Control group:63.9 ± 10.1
14	Marcus et al, 2010 (40)	USA	Hospital and medical Centre/NM	N=304 Age: <40 to >70 years
15	Hawkins et al, 2010 (41)	USA	Hospital and University based/NM	N=323 Age: Internet Access group: 52.3 ± 10.2; CHESS Group: 50.9 ± 9.0 Telephone group: 53.9 ± 10.9; CHESS ± Cancer information group: 52.7 ± 9.4
16	Baker et al, 2011 (42)	**USA**	Hospital/2005-2007	N=450 Age: Internet only group:52.3 ± 10.2; CHESS information only:52.2 ± 9.8; CHESS information and support:50.6 ± 0.8; Full Chess:50.9 ± 9.0
17	David et al., 2011 (74)	Germany	Medical University/2005-2008	N=235 Age: Intervention group: 48.2± 9.2; Control group:45.9 ± 7.8
18	Hawkins, 2011 (43)	USA	Hospital Cancer centres/NM	N=434 Age: Internet Only: 52.3 ± 10.2, Full Chess + Support + coaching: 50.9 ± 9.0; Mentor only: 53.9 ± 10.9; Full chess + Mentor: 52.7 ± 9.4
19	Hayes et al, 2011 (60)	Australia	University and hospital based/NM	N=194 Age: 52.4 ± 8.5
20	Hoyer et al, 2011 (69)	Denmark	Hospital/ 2010-2010	N=140 Age: Intervention group: 59 ± 9; Control group: 61 ± 8
21	Kimman et al, 2011 (66)	Netherland	Multi-center trial/NM	N=320 Age: Intervention group 56.2± 10.7 Control group: 55.5± 9.0
22	Sherman et al, 2011 (44)	USA	Medical centers and Hospital based/NM	N= 249 Age:53.8 ± 11.7
23	Crane-Okada et al, 2012 (45)	**USA**	Medical Institute/NM	N=142 Age: Immediate Contact group 63.4 ± 10.3; Delayed contact group(DC): 60.6 ± 7.4; Usual Contact group: 61.3± 8.7
24	Eakin at al, 2013 (61)	Australia	NM/2007-2009	N=143 Age: Intervention group: 51.7 ± 9.0; Control group: 54.1 ± 8.7
25	Hayes et al., 2013 (80)	Australia	University Hospital/2006 -2008.	N=194 Age: Face to face group:51.2 ± 8.8; Telephone group: 52.2 ± 8.6 Control group:53.9 ± 7.7
26	Pinto et al, 2013 (46)	USA	Hospital-based oncology clinic/2004-2009	N=192 Age: Intervention group: 56.1 ± 9.9; Control group: 55.9 + 9.9
27	Ryhanen et al., 2013 (77)	Finland	University Hospital/ 2008-2010	N=90 Age: Intervention group: 54.4; Control group: 55.7
28	Ziller et al, 2013 (75)	Germany	University Hospital/2006 -2008	N=181 Age: 63.3 ± 8.8
29	Goodwin et al, 2014 (78)	Canada	University based/ 2007-2010.	N=167: Age: Mail based intervention 60.4 ± 7.8; Individualized Lifestyle Intervention: 61.6 ± 6.7
30	Carpenter et al, 2014 (47)	USA	NM	N=115 Age: 50.9 ± 9.9
31	Berg et al, 2015 (65)	Netherlands	Multi Centre including University and hospitals/ 2010 -2012	N=150 Age: Intervention group:51.44± 8.30; Control group: 50.18 + 9.15
32	Freeman et al, 2015 (48)	USA	NM	N=118: Age: live-delivery 55.44 ± 8.08; Telemedicine group: 55.57 ± 9.88 Waitlist:55.28± 7.90.
33	Demark Wahnefried et al, 2015 (47)	USA	University based/2010-2012	N=697 Age: Intensive intervention group:56.0 ± 9.47; control group: 56.4 + 9.53
34	Befort et al, 2016 (49)	USA	Medical University/NM	N=172 Age: Intervention group:58.7 ± 8.2; Control group:57.3 ± 8.0
35	Chee et al, 2016 (50)	USA	NM/2014 -2015.	N=65 Age: Intervention Group: 46.1 ± 10.6; Control group: 48.0 ± 11.1
36	Damholdt et al, 2016 (14)	Denmark	University hospital/2013-2014.	N=157 Age: Intervention group: 54.98± 8.51; Control group: 54.56± 8.74
37	Galiano‐Castillo et al, 2016 (71)	Spain	Physical therapy lab in Health science faculty/2012-2013,	N=72 Age: Intervention group:47.4 ± 9.6 Control group: 49.2 ± 7.9
38	Harrigan et al, 2016 (51)	USA	Cancer institute/NM	N=100 Age: 59.0 ± 7.5 years
39	Abrahams et al, 2017 (67)	Netherland	Medical Centre 2014-2016	N=125 Age: Internet-based cognitive behavioral therapy (ICBT) group:52.5 ± 8.2;Care as Usual (CAU): 50.5 ± 7.6
40	Han et al, 2017 (52)	USA	Cancer institutions/2010-2014	N=560 Age: Intervention group: 45.8 ± 68.6 Control group: 46.4± 68.4
41	Gordon et al, 2017 (62)	Australia	University Hospital/ 2006-2008	N=194 Age: 52 ± 8
42	Bruggeman et al, 2017 (68)	Netherland	2013-2015	N=167 Age: Intervention group: 51.36 ± 12.04; Control group: 56.54 ± 8.43
43	Cox et al, 2017 (53)	USA	University based/NM	N=37 Age: Intervention group: 59.62 + 9.65; Control group: 59.92 ± 10.94
44	Zachariae et al, 2018 (70)	Denmark	NM/2011-2013	N=255 Age: Intervention group: 53.2 ± 8.8; Control group: 52.9 ± 8.9
45	Hartman et al, 2018 (55)	USA	2015- 2016 University based	N=87 Age:57.9+11.3
46	Kim et al, 2018 (79)	Korea	2013-2014/ University based	N=76 Age: Intervention group: 52.1; Control group: 49.8
47	Meneses et al, 2018 (22)	USA	Medical University	N=40 Age: 56.63 ± 10.63
48	Ferrante et al, 2018 (54)	USA	2016-2018 University based	N=37 Age:61.54 ± 8.83
49	Sherman et al, 2018 (63)	Australia	2015- 2015 University based	N=3014 Age: Intervention group: 57.5 ± 8.98; Control group:57.23 ± 9.97
50	Eun-Ok Im et al, 2019 (56)	USA	2017-2018	N=91 Age: 51.3 ± 11.31
51	Garcia et al, 2019 (72)	Spain	University based/NM	N=68 Age: Intervention group: 48.82 ± 7.68; Control group: 47.32 ± 9.92
52	Lynch et al, 2019 (64)	Australia	2016/NM	N=83 Age: Intervention group: 61.3 ± 5.9; Control group: 61.6 ± 6.4
53	Paladino et al, 2019 (57)	USA	2018-2021/ University based	N=200 Age: NM
54	Meneses et al, 2020 (58)	USA	NM/University based	N=432 Age: NM
55	Lleras de Frutos et al, 2020 (73)	Spain	2016-2019/NM	N=269 Age: Intervention group: 47.34 ± 8.05; Control group: 52.17 ± 8.36
56	Hou et al, 2020 (81)	Taiwan	2017-2018/University Hospital based	N=100 Age: Range (50-64 years)

**Table 3:** Interventions, outcome measures and results of included trials.

	Author/year/source	Intervention	Outcome measures/Assessment	Result
1	Rock et al, 2001 (29)	**T**elephone guided diet counseling	Average weight change % change, BMI, waist circumference Assessment: Baseline, 6 month, 12month,18month, 24 month	Diet intervention was not associated with significant weight loss
2	Samarel et al, 2002 (31)	Combined individual telephone and in person group support and education.	VAS-W, EWBS, a subscale of the Spiritual Well-Being Questionnaire UCLA Loneliness Scale–Version 3, Relationship Change Scale Assessment: Baseline, 13 month	A telephone based support intervention was found to be an effective option to in person support in early stage breast cancer survivors.
3	Pierce et al, 2004 (32)	Telephone counseling to promote dietary change	Dietary intakes, plasma carotenoid concentrations, Percentage energy from fat Assessment: Baseline,12 month	Telephone based counseling intervention promoted dietary change in breast cancer survivors.
4	Winzelberg et al, 2004 (33)	Structured, web based support group moderated by a mental health professional	*CES –D*, PCL-C54,STAI-55, PSS54CBI-55, Mini-MAC-58 Assessment: Baseline, 12 weeks	Web-based support group was found to be effective in reducing depression and cancer-related trauma, as well as perceived stress
5	Mishel, 2005 (34)	Telephone sessions for the use of audiotapes and self-help manual for behavioral strategies	Cancer Survivor Knowledge Scale, Patient/Provider Communication Scale, Social support satisfaction, CSQ, POMS-SF Assessment: Baseline,10 month	Improvement in cognitive reframing, coping skills, cancer awareness and communication in intervention group was observed
6	Owen et al, 2005 (76)	Internet-based group was given access to website for coping skills training exercises	QOL (FACT-B), Distress (IES) Assessment: Baseline, 12 week	Self-guided internet based coping technique resulted into improved self-rated health status and reduced distress.
7	Aranda et al, 2006 (59)	Face to face sessions and telephonic interactions for addressing concerns and coping strategies.	EORTC Q-C30, SCNS Assessment: Baseline,1 month and 3 month	Intervention significantly reduced the psychological and emotional needs of high needs group. However no effect was seen on low needs group
8	Gotay et al, 2007 (35)	Four to eight Telephonic calls over a 1-month period by trained peer counselors	CARES-S, CES-D Assessment: Baseline, 3 month,6 month	No statistically significant improvement was seen in distress and depression in telephonic counselling group.
9	Pierce et al, 2007 (30)	Telephone counseling regarding dietary intake.	Invasive breast cancer event (recurrence or new primary) or death from any cause Assessment: Baseline, 1year, 4 year	No significant reductions in cancer events or mortality was seen in telephone counselling group
10	Sandgren et al., 2007 (36)	Telephone-delivered health education	FACT-G, POMS, Revised PSS Assessment: Baseline, 6 month, 13 month	Telephone delivered sessions improved distress but no significant effect was seen on QOL.
11	Budin et al, 2008 (37)	Videos delivered psycho-education with telephonic counseling	PAIS, PAL-C, SRHS and the Breast Cancer Treatment Response Inventory Assessment: Baseline, diagnostic phase, 2 days post-surgery, 2 weeks after chemotherapy and 6 months post-surgery.	Intervention group had less distress and better psychological outcomes than standard care group
12	Kathleen et al, 2009 (38)	**T**elephone interview assessing adherence barriers; health education, problem-solving, and self-management support.	KPSS, FACT-G, Patient Health Questionnaire 9 Brief Symptom Inventory Assessment: Baseline, 12 month	Overall adherence rates range was good for both groups and no significant differences were noted.
13	Beaver et al, 2009 (39)	Telephone appointments to address questions related to changes in condition, new symptom development, required information about spread of disease, treatment and side effects, genetic risk, sexual attractiveness, self-care was provided.	STAI, GHQ-12, participants’ needs for information, participants’ satisfaction, clinical investigations ordered, and time to detection of recurrent disease. Assessment : Baseline,12 month	When compared to those who visited clinics in hospitals, participants in the telephone group showed less anxiety and higher levels of satisfaction. For women with a low to moderate recurrence risk, those with travel and movement issues, and those suffering from sickness with no physical or psychological disadvantage, telephone follow-up was found to be useful. In addition, the pressure on overburdened clinics was lessened.
14	Marcus et al, 2010 (40)	Telephone counseling program of 16 sessions for improving post treatment psycho social outcomes.	IES,CES-D, The Sexual Dysfunction scale Assessment: Baseline, 3 month,6 month, 12 month and 18 month	Telephone delivered counseling was found to be a viable option for providing psychological support to cancer survivors
15	Hawkins et al, 2010 (41)	Access to the Web-based comprehensive Health Enhancement Support System (CHESS), Telephone-based Cancer information	Health care competence, Cancer Information Competence, Emotional processing, Positive coping using Carver’s Brief Cope, FACT-B, Wisconsin social support scale Assessment: Baseline, 6 week	Combination of a computer-based information system and support produced significantly improved quality of life than for patients who were given training with general internet
16	Baker et al, 2011 (42)	Information, Support, and Coaching: Full CHESS. Training was conducted by telephone.	Cancer information outcomes, Health care competence, Emotional processing, positive coping, functional well-being, breast cancer concerns, satisfaction with professionals Assessment: baseline, 2 week, 6 week, 12 week, 24 week	E health interventions were found to be beneficial for survivors of breast cancer.
17	David et al., 2011 (74)	Email based individually tailored psycho education	EORTC QLQ-C30, BSI-GSI Assessment: Baseline, 2week	E mail based counseling was found to be beneficial for psycho-educational training of breast cancer survivors who are not being reached by conventional avenues of therapy. However, it may be difficult for patients with high distress level.
18	Hawkins, 2011 (43)	Access to the Web-based comprehensive Health Enhancement Support System (CHESS),Telephone-based Cancer information and mentorship	Functional well-being, emotional processing, social support and cancer information competence, breast cancer concerns, healthcare competence, satisfaction with professionals and positive coping. Assessment: Baseline, 6 week, 3 month, 6 month	On all the outcomes group with Full CHESS + Mentor group showed better scores than the Full CHESS condition.
19	Hayes et al, 2011 (60)	Telephone delivered 45 minutes of moderate-intensity physical activities including aerobic-based exercise, Strength-based exercise twice/week.	Breast (FACT-B+4) questionnaire Assessment: Baseline, 6 month,12 month	Participation of women during and after treatment was found to be feasible and acceptable.
20	Hoyer et al, 2011 (69)	Telephonic session by four experienced nurses for 10 to 30 minutes.	EORTC QLQ-C30 and EORTC QLQ-BR23 Assessment: Baseline, 2 week, 4 week	Telephone sessions did not bring statistically significant improvement in QOL of survivors.
21	Kimman et al, 2011 (66)	Nurse delivered telephone follow-up care and educational program	EORTC QLQ-C30, STAI Assessment: Baseline, 3 month, 6 month, 12 month	Nurse -led telephone follow-up can be an appropriate way to reduce number of visits to clinics and represents an accepted alternative strategy.
22	Sherman et al, 2011 (44)	Disease Management, standardized education and Telephone counselling	PAL-C, SRHS, PAIS, BCTRI Assessment: Baseline, 1 week before surgery,72 hours after surgery, 2 weeks, 6 month	The general finding for physical, emotional, and social adjustment is that normal care, which was the standard of treatment for women in both the control and intervention groups, supported their adjustment to breast cancer, with or without extra interventions.
23	Crane-Okada et al, 2012 (45)	Telephone based counseling sessions	HADS, IPRI, Short form social The Brief COPE Assessment: before surgery, post-intervention, and six months after surgery.	Peer counseling delivered by telephone may affect instrumental support seeking and appears to be differentially received by age group.
24	Eakin at al, 2013 (61)	Telephone delivered exercise intervention to increase women’s self-efficiency for exercise.	Feasibility indicators (recruitment and retention rates, sample representativeness, intervention implementation and participant satisfaction), Effectiveness outcomes were meeting intervention targets for aerobic and resistance training, quality of life, fatigue, anxiety and upper body function. Assessment: Baseline, 6 month,12 month	Results suggest strong support for feasibility and modest support for the efficacy of telephone-delivered interventions.
25	Hayes et al., 2013 (80)	Face to face and Telephone delivered exercise sessions (16)	FACT-B +4, fitness, functional status Assessment: Baseline, 6 month,12 month	Face to face or telephone delivered exercise intervention can prevent decline in fitness and function during treatment and optimize recovery post-treatment
26	Pinto et al, 2013 (46)	Telephone based counselling aimed to promote the level of physical activity	7-day PAR, Motivational Readiness for PA, MOS, SF-36, FACT-F Assessment: Baseline, 3 month, 6 month, 12 month	Telephone delivered counseling in addition to health care advise improved physical activity and readiness for physical activity in breast cancer survivors
27	Ryhanen et al., 2013 (77)	Internet-based patient educational program for empowerment of breast cancer patients	Instrument-Breast Cancer Patient Version, STAI Assessment: Baseline, 1 year	The internet delivered educational program did not decrease anxiety level or treatment-related side effects among breast cancer patients or improve subscales of quality of life when compared with controls
28	Ziller et al, 2013 (75)	Telephone delivered sessions to provide individualized information, feedback to questions and problems with medication	Self-reported adherence, MPR Assessment: Baseline, 12 month, 24 month	Groups that received additional information, improved adherence was seen however it was not statistically significant
29	Goodwin et al, 2014 (78)	Telephone-based intervention programme meant for weight reduction	Disease-free survival, Weight, overall survival, distant disease-free survival, quality of life Assessment: Baseline, 6 month, 12 month, 18 month, 24 month	A telephone based lifestyle intervention led to significant weight loss without adverse effects on QOL.
30	Carpenter et al, 2014 (47)	Online stress management workbook	IES, Revised CBI Assessment: Baseline, 10 week, 20 week	Internet based stress management therapy was helpful in reducing stress and improving confidence of breast cancer survivors
31	Berg et al, 2015 (65)	Web-based self-management intervention for reducing distress and improving empowerment.	EORTC QLQC30, IES, SES Assessment: Baseline,4 month	Access to web based management reduced distress among survivors, but this effect was not sustained during follow-up
32	Freeman et al, 2015 (48)	Live Delivery (LD) and Telemedicine delivered (TD) sessions (total five) 4-hour weekly group sessions, and received brief weekly phone calls to encourage at-home practice.	SF-36,FACT-B,FACIT-F, FACT-Cog, FACIT-Sp-Ex; version 4, BSIGSI,PSQI Assessment: Baseline, 1 month, 3 month	Telemedicine delivered intervention improved QOL and is recommended to be an alternative for cancer survivors specifically in remote areas
33	Demark Wahnefried et al, 2015 (47)	Weight loss program supplemented with telephone counseling and tailored newsletters.	Weight, IOCv2, SF-36,CES-D Assessment: Baseline, 6 month, 12 month, 2 year	There was improvement in some aspects of QOL in intervention group which diminished with time.
34	Befort et al, 2016 (49)	Telephone based counseling for weight loss, physical activity and weight loss maintenance.	Weight regain, Measures of weight change and costs. Assessment: Baseline 12 month	A lifestyle based intervention that included group phone-based support improved the intensity of weight loss, maintained and increased the proportion of survivors who maintained clinically significant reductions
35	Chee et al, 2016 (50)	**Internet** based Support was provided for emotional support, information and interaction.	FACT-B, CBI Assessment: pre-test, post test	Acceptance and satisfaction improved in intervention group.
36	Damholdt et al, 2016 (14)	Web-based cognitive training (e-CogT) with telephone support	Paced Auditory Serial Addition Test, Improvement on other measures of cognition. Assessment: Baseline, post-intervention and at 5-month follow-up.	Web-based cognitive therapy didn’t result in improvements in any of outcomes. Improved performance was observed on verbal learning and working memory
37	Galiano‐Castillo et al, 2016 (71)	Internet-based exercise intervention, videoconference and telephone calls	EORTC QLQC30 Assessment: Baseline, 6 month	Intervention group had significantly improved scores global health status, physical, role, cognitive functioning, and arm symptoms as well as pain severity and pain interference and muscle strength
38	Harrigan et al, 2016 (51)	Telephonic counselling regarding weight loss	Height and weight, Waist and hip circumference, Dual-energy x-ray absorptiometry scans, Physical activity, Number of steps walked/day, Change in daily calorie intake, serum biomarkers Assessment: Baseline, 6 month	Both telephonic and in person counseling were effective as weight loss strategy for breast cancer survivors
39	Abrahams et al, 2017 (67)	2 face to face sessions followed by online treatment (web modules) for which guidance was provided by cognitive behavioral therapist through e mail, telephone and video consultation.	Fatigue severity, Functional impairment, psychological distress, and quality of life. Assessment Baseline, 6 month	ICBT can be effective, evidence based and easily accessible treatment options for severely fatigued breast cancer survivors. However no effect was seen on QOL.
40	Han et al, 2017 (52)	An individually designed cancer-screening brochure, skills training, and telephone based counseling.	Psychosocial health outcomes Cancer information competence scale Assessment: Baseline, 6 weeks, 3 month and 6 month	Intervention group promoted cancer-screening behaviors and related cognitive and attitudinal outcomes
41	Gordon et al, 2017 (62)	Telephonic intervention.16 planned sessions by a trained exercise physiologist.	FACT-B+4 questionnaire; QALY’s and intervention costs Assessment: Baseline, 5 week, 6month,12 month	A combination of face to face and telephone based intervention resulted in improved QOL in breast cancer survivors.
42	Bruggeman et al, 2017 (68)	Web based mindfulness based cognitive therapy, accelerometer for feedback related to activity patterns	Fatigue severity, CIS-FS, HADS Assessment: Baseline, 2 week, 6 months	Both interventions were effective in reducing fatigue severity in both groups compared to group receiving psychoeducational mails
43	Cox et al, 2017 (53)	**Access** to online content by logging to website	Body composition, diet, physical activity, aerobic fitness Assessment: Baseline,6 month	Better health outcomes were seen in telephone group compared to internet group
44	Zachariae et al, 2018 (70)	Online CBTI (tele-education) program and completing sleep diaries	Sleep diary, insomnia severity by Insomnia Severity Index, PSI, and fatigue using FACIT-F Assessment: Baseline, 9 week, 15 week	Tele based CBTI programme resulted in improved sleep outcomes in survivors of breast cancer
45	Hartman et al, 2018 (55)	Wearable technology (fitbit) for self-monitoring of Physical activity	Physical activity measures Assessment: Baseline, 2 week, 3 week, 12 week	Technology based intervention helped survivors in tracking their physical activity levels
46	Kim et al, 2018 (79)	**M**obile game play group	Time spent for education, compliance to medical treatment, QOL, depression, anxiety Assessment: Baseline, 3 week	Patients who received an app-based intervention had better drug adherence, fewer chemotherapy side effects, and better patient education, but no effect on depression or anxiety, indicating the feasibility and potentiality of using smart phone mobile games for breast cancer patients receiving chemotherapy.
47	Meneses et al, 2018 (22)	Telephone education sessions, Support and early education	SF-36, CES-D Fatigue, pain Assessment: Baseline, 3 month, 6 month	Telephone based intervention helped in self-management of pain and fatigue
48	Ferrante et al, 2018 (54)	Physical activity tracking using technology (Fitbit)	Anthropometric measures, diet, Physical activity, cardiopulmonary fitness, QOL, body weight Assessment: Baseline, 1 month, 3 month	There was no significant effect on weight loss however improvement was seen in QOL, weight status, anthropometric measures and calorie intake
49	Sherman et al, 2018 (63)	Web based intervention to reduce stress	Body image related distress, body appearance scale, psychological distress and self-compassion Assessment: Baseline, 1 week, 1 month, 3 month	Web based intervention was helpful in reducing body image related distress, greater self-compassion and reduced psychological distress
50	Eun-Ok Im et al, 2019 (56)	Information and support with the help of mobile phones, computer and web based information	CBI-B, MSAS-SF Assessment: Baseline, 1 month, 3 month	Technology based intervention alleviated symptoms in survivors of breast cancer
51.	Garcia et al, 2019 (72)	Web based exercise intervention	6MWT, Fitness variables Assessment: Baseline, 8 week	A web based intervention helped in preventing decline in functional capacity and strength in breast cancer patients undergoing chemotherapy
52	Lynch et al, 2019 (64)	Wearable technology to assess physical activity levels as well as telephone delivered behavioral counselling	Physical activity levels and sedentary behavior Assessment: Baseline,12 week	Wearable technology may be a useful approach for breast cancer survivors to maintain an active lifestyle. There was an increase in physical activity and a decrease in sitting time.
53	Paladino et al, 2019 (57)	Received app based(web or internet) information about adherence to endocrinal treatment and feedback including links regarding coping strategies	Adherence to treatment, symptom management, FACT-ES,SF-12, PROMIS Assessment: Baseline, 6 month, 12 month	Intervention groups showed improved adherence to endocrinal treatment and self-management of symptoms
54	Meneses et al, 2020 (58)	Early education and support using telephone and mail	SF-36, CES-D, POMS, MOS-SSS Assessment: Baseline, 6 month	The use of a telephone-based intervention was found to be an effective way of reaching survivors in rural BC who were at risk of not receiving enough care.
55	Lleras de Frutos et al, 2020 (73)	Positive psychology classes were given *via* video-conferencing(online group)	HADS, PCL-C31, PTG1-34,CTB-R Assessment: Baseline, immediately after treatment, 3 months	Online positive psychology classes were effective in reducing distress in cancer survivors
56	Hou et al, 2020 (81)	Subjects received mobile health application based breast cancer self-management support	EORTC, QLQ-C30, QLQ-BR23	Mobile app based intervention was found to be effective in promoting QoL.

Center for Epidemiologic Studies Depression Scale (CES-D), Functional Assessment of Chronic Illness Therapy for Fatigue (FACIT-F), quality-adjusted life years (QALYs); The Short Form Health Survey (SF-36),The refined Impact of Cancer Scale (IOCv2), The Breast Cancer Prevention Trial (BCPT) Symptom Scales, Functional Assessment of Cancer Therapy-Breast (FACT-B), FACIT-Fatigue Scale (FACIT-F), FACT-Cog (version 2), Functional Assessment of Chronic Illness Therapy Spiritual Well-Being Expanded Scale (FACIT-Sp-Ex; version 4), 18-item Brief Symptom Inventory (BSI-18) Global Severity Index (BSIGSI), Pittsburgh Sleep Quality Index (PSQI), Medication possession ratio (MPR), Seven-Day Physical Activity Recall (7-day PAR), MOS 36-Item Short Form Health Survey (SF-36), Functional Assessment of Cancer Therapy Scale-Fatigue (FACT-F), Hospital Anxiety and Depression Scale (HADS), Interpersonal Relationship Inventory (IPRI), Functional Assessment of Cancer Therapy, Breast (FACT-B+4) questionnaire, Impact of Event Scale (IES),Epidemiologic Studies Depression Scale (CES-D), Adjustment to Illness Scale (PAIS), Profile of Adaptation to Life Clinical Scale (PAL-C), Self-rated Health subscale (SRHS), Cancer Rehabilitation Evaluation System–Short Form (CARES-SF), cognitive reframing subscale modified version of the cognitive coping strategies questionnaire (CSQ), European Organization of Research and Treatment of Quality of life Q-C30 version (2.0) (EORTC Q-C30) and Supportive Care Needs Survey (SCNS), PCL-C54,STAI-55, PSS54CBI-55, Mini-MAC-58, Visual Analogue Scale–Worry (VAS-W), Well-Being Scale (EWBS), MOS-SSS(Medical outcome study-social support survey, CIS-FS (Check individual strength fatigue scale), PCL-C31(Post traumatic stress disorder checklist version 31, PTGI-34(Post traumatic growth inventory), CTB-R (Revised cognitive therapy scale, PTGI-34(Post traumatic growth Inventory), FACIT-F(Facit fatigue scale), Functional Assessment of Chronic Illness Therapy Spiritual Well-Being Expanded Scale (FACIT-Sp-Ex; version 4), Brief Symptom Inventory (BSI-18) Global Severity Index (BSIGSI), Refined Impact of Cancer Scale (IOCv2), Cancer Rehabilitation Evaluation System–Short Form [CARES-SF], The European Organization for Research and Treatment of Cancer (EORTC), Quality-of-Life Questionnaire Core 30 (QLQ-C30),The EORTC Breast Cancer-Specific Quality-of-Life Questionnaire (QLQ-BR23), Quality of Life (QoL)

Varied outcome measures were evaluated in the trials. Studies targeting weight management in cancer survivors evaluated weight status, calorie intake and Body Composition. Studies that assessed psycho behavioral aspects used different outcomes like depression, anxiety, sleep and sexual dysfunctions, spiritual and emotional wellbeing, psychological morbidity, self-reported functional status, adjustment to life and adherence to treatment. Studies that examined effects of exercise interventions evaluated Physical activity status, quality of life, self-related health outcomes and functional status. Recurrence of cancer and death was also evaluated in 1 study by Pierce et al, 2007 (30). Interventions and outcome measures are presented in **Table 3**.

### Risk of bias

Four trials were judged with high risk of bias in the domain of random sequence generation (33, 38, 47, 74), as methods of randomization were not given in detail. Twenty trials were judged with low risk of bias in the domain of allocation concealment (29–32, 38, 39, 42, 44, 51, 54, 60, 63–65, 67, 69, 71–73, 80). Eleven studies reported blinding of participants and personnel (14, 29, 32, 38, 42, 51, 63, 64, 72, 75, 80) while twelve trials mentioned about blinding of outcome assessors (38, 39, 42, 51, 60, 64, 67, 69–72, 80) and hence were regarded at low risk of bias. Six studies were reported at high risk in the domain of incomplete outcome data (35, 39, 56, 61, 62, 73). Therefore, in future researches, the allocation concealment, blinding of participants, personnel and outcome assessors should be emphasized to bring out better and reliable conclusions. Risk of bias is presented in **Table 4**.

**Table 4:** Risk of bias assessment.

Trial	Random sequence generation	Allocation concealment	Blinding of patient and personnel	Blinding of outcome assessment	Incomplete outcome data addressed	Selective reporting	Other Bias
1. Rock et al, 2001	Low	Low	Low	Some concern	Low	Low	Low
2. Samarel et al, 2002	Low	Low	High	High	Low	Low	Low
3. Pierce et al, 2004	Low	Low	Low	Some concern	Low	Low	Low
4. Winzelberg et al., 2003	High	High	High	High	Low	Low	Low
5. Mishel, 2005	Low	High	High	High	Low	Low	Low
6. Owen. 2005	Low	High	Some concern	High	Low	Low	Low
7. Aranda et al, 2006	Low	Some concern	Some concern	Some concern	Low	Low	Low
8. Gotay et al, 2007	Low	High	High	High	High	Low	Low
9. Pierce et al, 2007	Low	Low	High	Some concern	Low	Low	Low
10. Sandgren et al, 2003	Some concern	Some concern	High	High	Low	Some concern	Low
11. Budin et al, 2008	Some concern	Some concern	Some concern	Some concern	Low	Low	Low
12. Kathleen et al, 2009	High	Low	Low	Low	Low	Low	Low
13. Beaver et al, 2009	Low	Low	Some concern	Low	High	Low	Low
14. Marcus, 2010	Yes	High	High	High	Low	Low	Low
15. Hawkins, 2010	Low	High	High	High	Low	Low	Low
16. Baker et al, 2011	Low	High	High	High	Low	Low	Low
17. David et al, 2011	High	High	High	High	Low	Low	Low
18. Hawkins, 2011	Low	Some concern	Some concern	High	Low	low	Low
19. Hayes et al, 2011	Low	Low	Some concern	Low	Low	Low	Low
20. Hoyer, 2011	Low	Low	High	Low	Low	Low	Low
21. Kimman et al, 2011	Low	Some concern	High	Some concern	Low	High	Low
22. Sherman et al, 2011	Low	Low	High	High	Low	Low	Low
23. Crane-Okada et al, 2012	Low	High	High	Some concern	Low	Low	Low
24. Eakin et al, 2013	Low	Some concern	Some concern	Some concern	High	Low	Low
25. Hayes, 2013	Low	Low	Low	Low	Low	Low	Low
26. Pinto et al, 2013	Low	High	High	High	Low	Low	Low
27. Rhyanen et al, 2013	Low	Some concern	Some concern	Some concern	Low	Some concern	Low
28. Ziller et al, 2013	Low	Some concern	Low	Some concern	Low	Low	Low
29. Goodwin et al, 2014	Low	Some concern	Some concern	High	Low	Low	Low
30. Carpenter. 2014	Low	High	Some concern	High	Low	Low	Low
31. Berg, 2015	Low	Low	Some concern	Some concern	Low	Low	Low
32. Freeman et al, 2015	Low	Some concern	High	Some concern	Low	Low	High
33. Demark Wahnefried et al, 2015	High	Some concern	Some concern	High	Low	Low	Low
34. Befort et al, 2016	Low	Some concern	High	Some concern	Low	Low	Low
35. Chee, 2016	Low	High	High	High	Low	Low	Low
36. Damholdt et al, 2016	Low	Some concern	Low	Some concern	Some concern	Low	Low
37. Galiano Castilo et al, 2016	Low	Low	Some concern	Low	Low	Some concern	Low
38. Harrigan et al, 2016	Low	Low	Low	Low	Some Concern	Low	Low
39. Abrahams et al,2017	Low	Low	High	High	Low	Low	Low
40. Han et al, 2017	Low	Some concern	High	High	Low	Low	Low
41. Gordon et al, 2016	Low	Some concern	Some concern	Low	High	Low	Low
42. Bruggeman et al, 2017	Low	High	High	Low	Low	Low	Low
43. Cox et al., 2017	Low	High	High	High	Low	Low	Low
44. Zachariae et al., 2018	Low	High	High	High	Low	Low	Low
45. Hartman et al., 2018	Low	High	High	High	Low	Low	Low
46. Kim et al., 2018	Low	High	High	High	Low	Low	Low
47. Meneses et al., 2018	Some concern	High	High	High	Low	Low	Low
48. Ferrante et al., 2020	Low	Low	High	High	Low	Low	Low
49. Sherman et al., 2018	Low	Low	Low	Some concern	Low	Low	Low
50. Eun-Ok Im et al,2019	Some concern	High	High	High	High	Low	Low
51. Garacia et al., 2019	Low	Low	Low	Low	Low	Low	Low
52. Lynch et al., 2019	Low	Low	Low	Low	Low	Low	Low
53. Paladino et al., 2021	Low	High	High	High	Low	Low	Low
54. Meneses et al., 2020	Some concern	Some concern	High	High	Low	Low	Low
55. Lleras de Frutos et. al., 2020	Low	Low	High	High	High	Low	Low
56. Hou et al., 2020	Low	Low	Low	Low	Low	Low	Low

### Treatment Outcomes

#### Meta-analysis of depression

**Figure 2:**
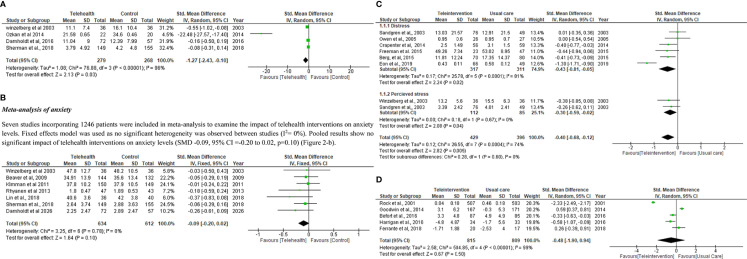
**(A)** Meta-analysis of depression. **(B)**Meta-analysis of anxiety.**(C)** Meta-analysis of **(A)** Distress **(B)** perceived stress. **(D)** Meta-analysis of weight change. **(E)** Meta-analysis of Quality of life.

Four trials with 547 participants reported the outcomes of depression in meta-analysis. Random-effects model was used due to significant heterogeneity across these trials (I^2 =^ 96%, Tau^2 =^ 1.08). Pooled results indicated that telehealth intervention were associated with reduced depression levels in breast cancer patients (SMD -1.27, 95% CI =-2.43 to -0.10 p=0.03) (**Figure 2A**).

#### Meta-analysis of anxiety

Seven studies incorporating 1246 patients were included in meta-analysis to examine the impact of telehealth interventions on anxiety levels. Fixed effects model was used as no significant heterogeneity was observed between studies (I^2 =^ 0%). Pooled results show no significant impact of telehealth interventions on anxiety levels (SMD -0.09, 95% CI =-0.20 to 0.02, p=0.10) (**Figure 2B**).

#### Sub group analysis of distress and perceived stress

Six studies involving 628 patients were included in meta-analysis to determine the impact of telehealth interventions on distress. Random effects model was used as high heterogeneity was observed among studies (I^2 =^ 81%). Pooled results depict that a significant impact of telehealth interventions was observed on distress (SMD -0.27, 95% CI =-0.44 to -0.09, p=0.003) (**Figure 2C**).

Subgroup analysis including 825 patients was carried out to determine the impact of telehealth interventions on perceived stress and distress levels. Random effects model was used as high heterogeneity was observed among studies (I^2 =^ 74%). Six studies involving 628 patients were included to determine the impact of telehealth interventions on distress while two studies including 197 patients were included to determine the impact of telehealth interventions on perceived stress. Pooled results depict that a significant impact of telehealth interventions was observed on distress and perceived stress levels (SMD -0.40, 95% CI =-0.68 to -0.12, p=0.005) (**Figure 2C**).

#### Dietary status and weight change

### Meta-analysis of weight change

Five studies incorporating 1624 subjects were incorporated in the meta-analysis of weight change. Random effects model was used due to more heterogeneity among studies (I^2 =^ 99%). Pooled results depict that no significant impact of telehealth interventions was observed on weight change levels also (SMD -0.48, 95% CI =-1.90 to 0.94, p=0.50) (**Figure 2D**).

### Quality of Life

#### Meta-analysis of Quality of life

Seventeen RCTs including 3055 breast cancer patients were included in the meta-analysis of QOL. Different QOL measurement scales reported in these trials are: FACT G, EORTC QLQ-C30, SF36, FACT-B, FACT-B+4, BCPT and Impact of Cancer Scale. Standardized mean difference (SMD) was used because of variety of measurement scales used in trials. Pooled results depict that telehealth interventions significantly improved the QOL score in breast cancer patients (SMD 0.48, 95% CI 0.03 to 0.92, p=0.04) (**Figure 2E**).

## Discussion

In recent decades, medical technology has experienced significant development (82). In addition, breast cancer patients nowadays tend to have better survival rates compared with those in the past. However, during the survival period, these patients’ QOL, physical and psychological health need close attention. Psychological symptoms such as sadness, anxiety and perceived stress are common and generally untreated in breast cancer patients, which can have a detrimental impact on their quality of life. Also, physical health issues like weight gain and obesity can result into recurrent risk, poor prognosis and all-cause mortality in breast cancer survivors (30, 83). Lifestyle interventions in form of weight reduction has been recommended to improve health outcomes (84). In comparison to traditional care, telehealth is a highly accessible and effective intervention that may overcome time and location obstacles. Patients can connect with medical professionals about their disease issues and gain more information about disease management through telehealth care. These situations can give patients with continual access to assistance and make them feel that they’re not alone and that medical help is always nearby both of which are advantageous to their psychological well-being. The use of telehealth has numerous advantages for breast cancer patients, but there are also many challenges and issues among patients, healthcare professionals, and service providers. These include patient’s unwillingness to use the technology, especially older patients who prefer in-person consultations, inconsistent internet connections in rural regions, patient mistrust because a thorough physical examination cannot be performed remotely, and inadequate insurance coverage. Additional challenges to telehealth include concerns regarding the security of patient health records transmitted electronically, high acquisition and implementation costs, significant maintenance costs, management and training of healthcare professionals to effectively use the various platforms and limited access to technology or low platform literacy. To the best of our knowledge, this study represents the first meta-analysis to examine the effect of telehealth intervention from inception till date on various physical and psychological health parameters in breast cancer patients. The results revealed that compared with usual care, telehealth intervention was associated with higher QOL, with less depression, distress and perceived stress symptoms however no significant effect was seen on anxiety and weight status. Fifty Six RCTs incorporating telehealth modalities for breast cancer women were included in this review. Telephone was found to be the leading telehealth tool in most of the studies. A large number of studies also supported use of web based interventions for various physical and psychological outcomes in cancer survivors. There has been an increasing interest in the use of smart wearable technologies to encourage breast cancer survivors to modify their physical activity (PA) habits. Alternate telehealth technologies like mobile-based apps or other advanced e-Health systems have also seen an upsurge in last few years. A precise, reproducible, trustworthy, and affordable diagnosis of breast cancer lymphedema can be made using augmented reality techniques, such 3DLS, in the clinical setup (84). However more number of RCT’s are needed to evaluate their efficacy on weight status, QOL and mental health parameters. Majority of telehealth interventions were related to awareness using educational/supportive material based on scheduled phone calls aimed at improving physical and psychological health of study populations. To enhance the quality of this systematic review, only randomized controlled trials were included and quality was assessed using the Cochrane risk of bias tool. Timely information and consultation with experts is a crucial aspect for women suffering from breast cancer. Technological advancements have improved the survival rates of these patients. But, during the survival period, their health parameters need to be vigilantly monitored. Our findings are consistent with previous studies that show that breast cancer patients need continuous consultation that would help them in understanding their condition better so that they can cope with the treatment process more confidently (83, 85, 86). The results of this systematic review indicate that telehealth technologies could considerably improve quality of life, physiological and psychological parameters of breast cancer patients.

The increasing enthusiasm for tele health is determined not only by its established benefits, but also by the extensive accessibility of mobile phones, and the comparatively low levels of education required to use them (1).In comparison to traditional care, telehealth is a highly accessible and effective intervention that may overcome time and location obstacles (65). Patients can conveniently interact with health professionals about their medical conditions and get more information about disease management through telehealth care (87). Results of our review also show majority of trials used telephone based interventions. The dominance of telephone based and Web-based telehealth interventions makes participant recruitment easy and facilitates timely data collection. Also the risk of missing information is reduced and follow up becomes easy. Furthermore, eHealth interventions are relatively more cost effective and provide wide geographical coverage overcoming mobility issues. But researchers have less control over respondents (77, 88). The COVID-19 pandemic has significantly transformed how healthcare is provided. In order to sustain patient care while reducing the danger of nosocomial SARS-COV-2 infection, decentralization measures such telehealth visits, home-based care, and remote patient monitoring should be quickly adopted. These techniques can be used to relieve the burden of treatment and lower the risk of exposure for patients and medical staff across the entire spectrum of care, from prevention to palliation (89–92). Moreover, our findings also divulge that telehealth interventions are primarily used in developed nations while their use in developing countries is still less. This may be due to inappropriate resource allocation, dearth of technical expertise, high initial investment and deficient healthcare infrastructure in developing countries.

The large scale search conducted in multiple databases, inclusion of exclusive randomized controlled trials, methodological quality assessment are the strengths of this review. Studies that had only telehealth interventions were included thus making comparison of studies feasible. Another strength is inclusion of wide range of physical, physiological and psychological outcome measures. There are some limitation also. Differences between duration of interventions, outcomes measures and varied control groups in trials led to heterogeneity. Also, inclusion of trials written in English language only was another limitation that may introduce publication bias.

## Conclusion

This systematic review concludes that telehealth care is a quick, convenient and assuring approach to breast cancer care in women that can reduce treatment burden and subsequent disturbance to the lives of breast cancer survivors. Telehealth interventions are worthy of clinical consideration and should be used as part of a holistic breast cancer treatment plans. We suggest that additional resources should be placed in the development of telehealth care and more high-quality randomized controlled trials should be conducted to investigate the worth of telehealth care in the management of breast cancer patients. It is also important to tailor and develop telehealth interventions according to survivor’s needs, possibly by involving them in the early stages of intervention design to curtail perception of impersonal care and attain benefits of remote monitoring.

## Data availability statement

The original contributions presented in the study are included in the article/supplementary material. Further inquiries can be directed to the corresponding author.

## Author contributions

Conceptualization: Done by PA, SK, RG. Designing the study: RG, VC, RS. Data collection: PA, MA, AA, MAI, SM, SP. Compilation, analysis and interpretation of data: PA, RA, VM, MM. Manuscript writing and review: MM, PA, SK, VM. All the drafts were reviewed by PA, MM, SK, RG, VC, RS, MA, AA, MAI, SM. All authors contributed to the article and approved the submitted version.
